# Primary intracranial Ewing-like sarcoma presenting with rapid bilateral blindness: A case report

**DOI:** 10.1016/j.radcr.2026.05.011

**Published:** 2026-06-02

**Authors:** Mohammed Amine Guerrouaz, Soufiane Berhili, Mohamed Moukhlissi, Karim Bahhous, Loubna Mezouar

**Affiliations:** Radiation Oncology Department, Mohammed VI University Hospital Center, Oujda, Morocco

**Keywords:** Intracranial Ewing sarcoma, Primitive neuroectodermal tumor, Brain tumor, MRI, Vision loss

## Abstract

Primary intracranial Ewing sarcoma / Ewing-like sarcoma is an exceptionally rare malignant small round cell tumor. Its radiological appearance is often non-specific and may mimic other aggressive intracranial neoplasms, leading to diagnostic delay. We report the case of a young adult presenting with progressive headaches and recurrent generalized tonic–clonic seizures for six months, followed by rapid bilateral visual loss. Neurological examination revealed papilledema and decreased visual acuity, prompting brain imaging. MRI demonstrated a large aggressive frontal mass with heterogeneous enhancement, marked diffusion restriction, meningeal involvement, and significant mass effect with midline crossing. Initial staging showed no extracranial disease. Histopathology revealed a small round cell tumor with strong membranous CD99 expression consistent with an intracranial Ewing-like sarcoma. The patient received multimodal treatment including radiotherapy and VDC/IE chemotherapy. Follow-up imaging demonstrated partial tumor response. However, no visual recovery occurred, and the patient remained limited to light and shadow perception. This case highlights the diagnostic challenges of primary intracranial Ewing-like sarcoma and its potential to present with severe and irreversible visual impairment. Early neuroimaging in patients with persistent seizures is essential to avoid diagnostic delay, and molecular confirmation remains crucial for future cases.

## Introduction

Intracranial involvement in Ewing sarcoma is extremely rare. In a large international retrospective study including 1435 patients with Ewing sarcoma, only 21 cases (1.5%) presented with intracranial disease at diagnosis, among which 17 cases (1.2%) were primary intracranial tumors [1]. These findings highlight the exceptional rarity of primary intracranial Ewing sarcoma and the limited clinical experience available in the literature.

Because of this rarity, diagnosis and management remain challenging and require a multidisciplinary approach integrating clinical findings, imaging, histopathology, and molecular analysis.

## Case report

A 29-year-old female presented with a six-month history of progressive headaches and multiple generalized tonic-clonic seizures.

During the first months, seizures were managed symptomatically in a primary care setting, where they were initially considered idiopathic epilepsy due to the absence of focal neurological deficits. Neuroimaging was performed once neurological symptoms progressed and visual disturbances appeared, prompting referral for specialized neurological assessment.

Neurological examination revealed papilledema, decreased visual acuity, and signs of intracranial hypertension, which prompted urgent brain computed tomography (CT) scan, which demonstrated a left frontoparietal space-occupying lesion, partially calcified and appearing extra-axial, associated with significant mass effect and surrounding vasogenic edema.

However, subsequent Magnetic resonance imaging MRI analysis demonstrated predominantly intra-axial characteristics left frontal lesion involving both cortical and subcortical regions measuring 57 × 70 mm with an anteroposterior extent of 76 mm. The lesion demonstrated mild hyperintensity on T2-weighted and FLAIR sequences and marked diffusion restriction within the solid tumor component with low ADC values compared with normal white matter, reflecting high tumor cellularity. Heterogeneous gadolinium enhancement was observed with areas of necrosis and calcifications. Marked peritumoral edema caused compression of the ipsilateral lateral ventricle, midline shift, and early signs of subfalcine herniation. The tumor infiltrated the midline with partial extension into the contralateral frontal lobe while sparing the skull base inferiorly and demonstrating associated meningeal involvement laterally without evidence of calvarial bone destruction (Fig. 1). Overall, the lesion can be summarized radiologically as a large predominantly intra-axial frontal mass with heterogeneous signal intensity, marked diffusion restriction, necrotic components, heterogeneous contrast enhancement, extensive vasogenic edema, and significant mass effect with midline crossing.

**Fig. 1:**
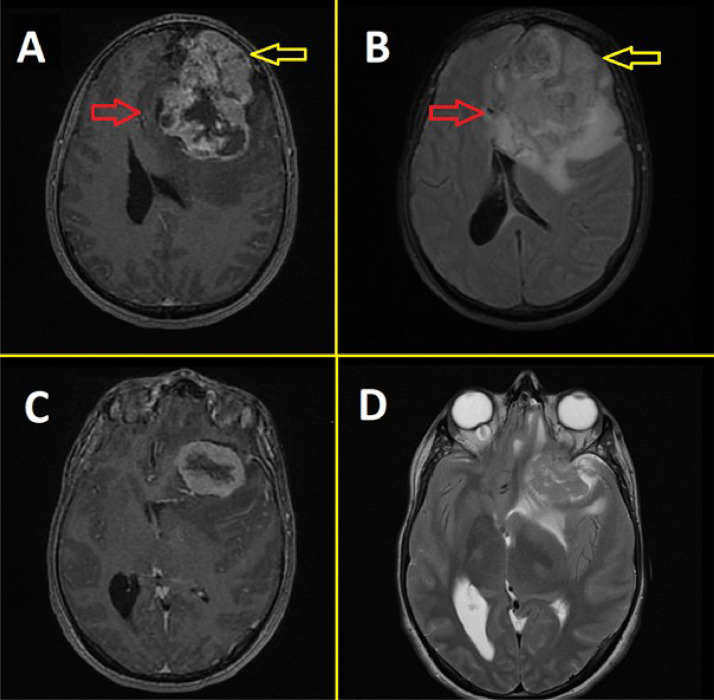
Brain MRI showing a large left frontal predominantly intra-axial mass with heterogeneous enhancement, necrotic areas, extensive edema, and midline crossing (red arrow). Meningeal involvement is visible without calvarial bone destruction (yellow arrow).

A staging cervico-thoraco-abdomino-pelvic CT scan showed no evidence of extracranial lesions. The patient declined surgical resection; therefore, a stereotactic biopsy was performed.

Histopathological examination revealed a poorly differentiated small round cell tumor composed of densely packed malignant cells. Immunohistochemistry demonstrated strong membranous expression of CD99 and negative staining for GFAP, cytokeratin AE1/AE3, desmin, S100, and CD45. The Ki-67 proliferative index was approximately 50%. These findings supported the diagnosis of an intracranial Ewing-like sarcoma.

The multidisciplinary tumor board recommended radiotherapy targeting the cerebral lesion followed by systemic chemotherapy using the VDC/IE protocol.

The patient subsequently underwent radiotherapy. The gross tumor volume (GTV) was defined based on the planning CT scan fused with brain MRI. A 1-cm margin was added to generate the clinical target volume (CTV), followed by an additional 3-mm margin to obtain the planning target volume (PTV). A total dose of 58 Gy in 29 fractions was delivered while respecting organs at risk, particularly the brainstem (Fig. 2).

**Fig. 2:**
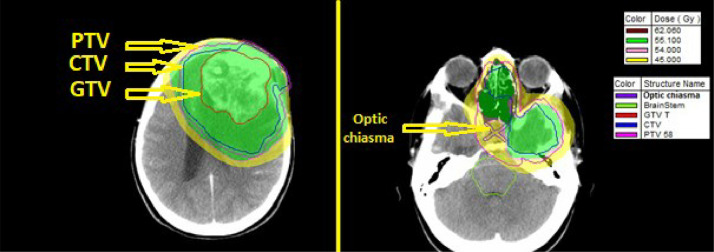
Axial radiotherapy planning CT demonstrating target volume delineation following fusion with brain MRI. The gross tumor volume (GTV) is shown in red, the clinical target volume (CTV) in blue, and the planning target volume (PTV) in pink. Organs at risk include the brainstem (green) and the optic chiasm (purple). demonstrating isodose lines covering the planning target volume (PTV) while sparing surrounding organs at risk, including the brainstem and optic chiasm.

A follow-up cervico-thoraco-abdomino-pelvic CT scan was performed after radiotherapy to reassess systemic staging and exclude interval metastatic spread, given the aggressive nature of the tumor and the high metastatic potential of Ewing sarcoma family tumors and demonstrated a slight reduction in tumor size to 48 × 69 × 65 mm compared with the initial 57 × 70 × 76 mm, consistent with a partial tumor response and without evidence of metastatic disease (Fig. 3).

**Fig. 3:**
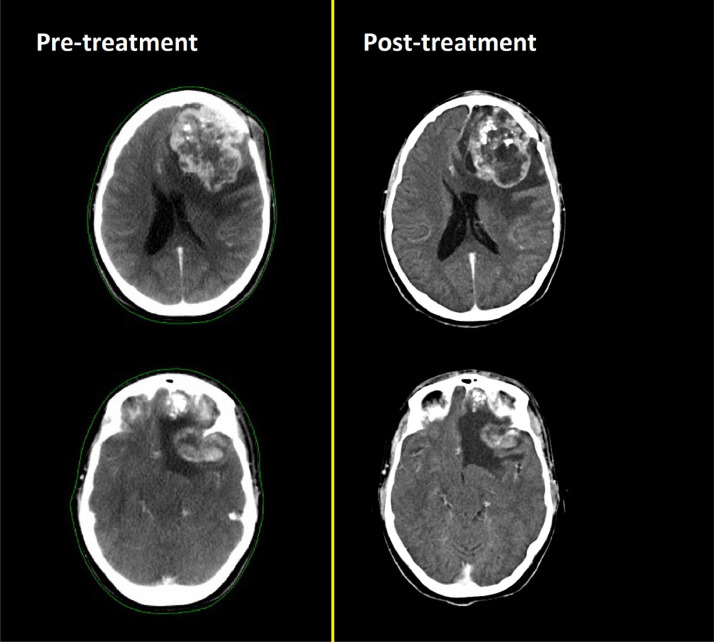
Post-treatment CT scan showing partial reduction of the intracranial tumor size following radiotherapy with no distant metastases detected.

Systemic chemotherapy using the VDC/IE regimen (vincristine, doxorubicin, cyclophosphamide alternating with ifosfamide and etoposide) was subsequently initiated. After six cycles, follow-up brain MRI showed further tumor reduction to 42 × 62 × 59 mm with decreased perilesional edema, consistent with a partial response (Fig. 4). The patient is currently continuing treatment.

**Fig. 4:**
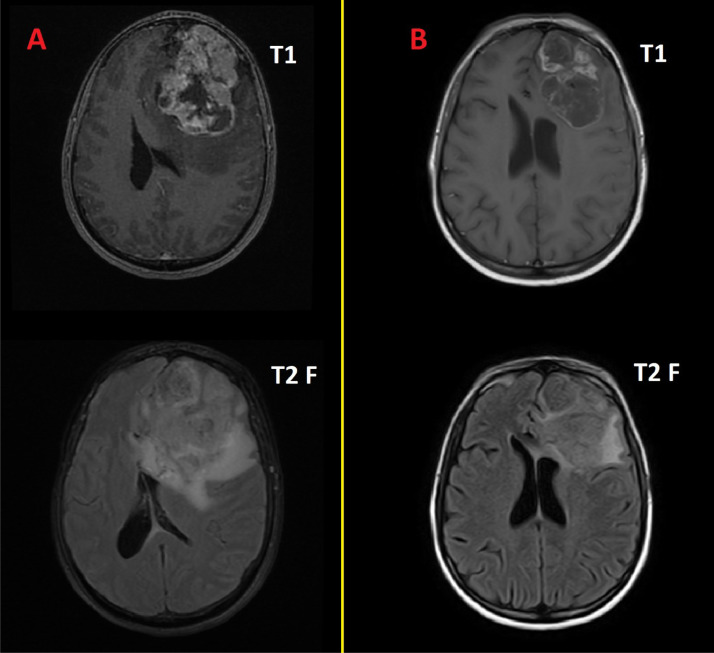
Follow-up MRI after six cycles of VDC/IE chemotherapy and completion of radiation therapy demonstrating further tumor reduction.

Regarding visual outcome, no visual improvement was observed during follow-up. The patient remained limited to light and shadow perception, consistent with persistent severe visual impairment.

## Discussion

Primary intracranial Ewing sarcoma remains a diagnostically and therapeutically challenging entity because of its rarity and the absence of well-established management guidelines. Most available evidence derives from small case series and isolated reports, which makes every additional case valuable for improving recognition of clinical presentation, imaging spectrum, and treatment strategies.

The diagnosis of small round-cell sarcomas relies on a combination of histopathology, immunohistochemistry, and molecular testing. Classical Ewing sarcoma is composed of uniform small round cells with scant cytoplasm and round nuclei with finely dispersed chromatin, typically showing strong, diffuse membranous CD99 expression and nuclear NKX2.2 positivity [2]. Ewing-like sarcomas share similar morphology but harbor distinct molecular alterations, such as CIC–DUX4 rearrangements with variable CD99 and ETV4 or WT1 expression, or BCOR–CCNB3 fusions with BCOR, SATB2, and CCNB3 positivity [2,3]. In the present case, molecular analysis was not performed. Histopathology showed densely packed small round cells with hyperchromatic nuclei and scant cytoplasm. Immunohistochemistry revealed strong, diffuse membranous CD99 positivity, with tumor cells negative for GFAP, S100, desmin, cytokeratin, and CD45. This profile excluded lymphoma, glial tumors, rhabdomyosarcoma, and metastatic carcinoma. Combined with morphological features and clinical–radiological context, these findings support the diagnosis of an intracranial Ewing-like small round cell sarcoma. Because molecular testing for EWSR1 gene rearrangement was not available in our institution, the diagnosis could not be molecularly confirmed. Therefore, we deliberately use the descriptive term “Ewing-like sarcoma” based on histopathology and strong membranous CD99 expression. This represents an important diagnostic limitation of the present report [4].

## Our case particularity : Blindness

In a retrospective study including 240 children with brain tumors, it is reported that severe irreversible visual loss occurred in approximately 5% of patients and was most often related to optic nerve compression or papilledema secondary to increased intracranial pressure. Importantly, many patients already had significant visual impairment at the time of diagnosis, emphasizing the importance of early detection and treatment to prevent permanent optic nerve damage [5].

Similarly, a cohort of 18 patients in whom visual disturbances were the primary manifestation of intracranial tumors, including visual loss, visual field defects, and transient visual obscurations. In that series, the mean delay between the onset of visual symptoms and tumor diagnosis was nearly ten months, illustrating how visual complaints may represent the first clinical manifestation of intracranial neoplasms and should prompt early neuroimaging evaluation [6].

In our patient, rapidly progressive visual loss leading to bilateral blindness was one of the major presenting symptoms.

In our case, imaging did not demonstrate direct optic nerve compression. Visual loss was therefore most likely related to severe intracranial hypertension and papilledema secondary to the large tumor and associated mass effect. Despite tumor response to treatment, no visual recovery occurred, and the patient remained limited to light and shadow perception, highlighting the risk of irreversible optic pathway damage when diagnosis is delayed.

In the present case, imaging demonstrated a large aggressive predominantly intra-axial frontal mass with heterogeneous enhancement, necrotic components, and extensive vasogenic edema producing marked mass effect and midline shift. The tumor also showed meningeal involvement without evidence of calvarial bone destruction.

Historically, intracranial Ewing sarcoma has most commonly been described as an extra-axial or dural-based lesion mimicking meningioma [7,8]. However, more recent reports increasingly recognize true intra-axial intraparenchymal presentations that may closely resemble high-grade gliomas [7,9]. Our case fits within this less common intra-axial phenotype and additionally demonstrates midline crossing, an imaging feature that remains particularly unusual.

Calvarial involvement in intracranial Ewing sarcoma appears to be variable. While some cases demonstrate skull destruction with extracranial extension, others—such as the present case—show aggressive intracranial disease without bone erosion [10]. This observation is clinically important, as the absence of bone involvement should not exclude the diagnosis.

Because of its nonspecific radiologic appearance, intracranial Ewing sarcoma is frequently misdiagnosed. The principal differential diagnoses include high-grade glioma, primary CNS lymphoma, aggressive meningioma, metastasis and intracranial small round cell tumors. Features such as marked diffusion restriction reflecting high cellularity, heterogeneous enhancement, necrosis, and extensive edema contribute to this diagnostic overlap [[6], [7], [8]]. Consequently, imaging alone remains insufficient for definitive diagnosis, and histopathological confirmation is essential.

Magnetic resonance spectroscopy, although not available in the present case, may provide additional metabolic information. Small round cell tumors typically demonstrate elevated choline levels, reduced N-acetylaspartate, and occasional lipid peaks reflecting high cellular turnover and necrosis [11].

A limitation of this report is that whole-spine MRI was not performed. Although staging CT showed no extracranial disease, subclinical cerebrospinal fluid dissemination cannot be completely excluded.

Historically, the vincristine–actinomycin D–cyclophosphamide (VAC) regimen was considered the standard chemotherapy backbone for Ewing sarcoma for several decades. Early cooperative studies, including the Intergroup Ewing's Sarcoma Study, demonstrated that multi-agent chemotherapy combined with local treatment significantly improved outcomes. In particular, a previous study reported that local tumor control in Ewing sarcoma was influenced by radiation dose, treatment volume, and primary tumor site, highlighting the importance of optimized radiotherapy planning in local disease control [12].

Treatment strategies evolved after the landmark randomized trial published in the New England Journal of Medicine by Grier et al., which demonstrated that the addition of ifosfamide and etoposide to standard chemotherapy significantly improved outcomes in patients with localized Ewing sarcoma. In that study involving 518 patients, the intensified regimen achieved a 5-year event-free survival of 69% compared with 54% with standard therapy [13].

More recently, the EE2012 phase III trial conducted by Brennan et al. compared two chemotherapy schedules in newly diagnosed Ewing sarcoma. The study showed that the interval-compressed VDC/IE regimen administered every two weeks significantly improved event-free survival compared with the European VIDE regimen. These results support VDC/IE as the preferred induction chemotherapy regimen for Ewing sarcoma [14].

Ewing-like sarcomas represent a heterogeneous group of undifferentiated round cell sarcomas that share morphological similarities with Ewing sarcoma but harbor distinct molecular alterations, including CIC or BCOR rearrangements. Due to their rarity and the lack of prospective clinical trials, management generally follows therapeutic strategies used for classical Ewing sarcoma and relies on a multimodal approach combining systemic chemotherapy with surgery and/or radiotherapy [2].

This case also highlights that non-surgical management may achieve meaningful tumor control in selected patients, particularly when surgical resection is not feasible or declined. Our patient achieved a sustained partial response with radiotherapy followed by VDC/IE chemotherapy, supporting the role of multimodal therapy even in the absence of surgery.

Intracranial involvement in Ewing sarcoma is extremely rare. In the retrospective international study, patients with primary intracranial Ewing sarcoma treated with multimodal therapy demonstrated a 3-year overall survival of approximately 70%. In contrast, patients with intracranial metastatic disease had significantly poorer outcomes, reflecting the aggressive nature of disseminated disease [1].

Another important prognostic factor is the histopathologic response to induction chemotherapy. In a landmark study, it was demonstrated that patients achieving ≥90% tumor necrosis after neoadjuvant chemotherapy had significantly improved survival compared with poor responders. These findings highlight the critical role of systemic therapy and treatment response in determining long-term outcomes in patients with Ewing sarcoma [15].

## Conclusion

Primary intracranial Ewing-like sarcoma is an exceptionally rare and aggressive tumor that may present with atypical manifestations such as rapid bilateral blindness. Imaging findings are often nonspecific and may mimic high-grade glioma, lymphoma, or aggressive meningioma, making histopathological confirmation essential. Although our patient achieved partial response with radiotherapy followed by VDC/IE chemotherapy, the absence of molecular confirmation represents a diagnostic limitation. Reporting such cases contributes to improved recognition of this rare entity and supports the role of multidisciplinary management.

## Patient consent

Written informed consent was obtained from the patient for publication of this case report and any accompanying images.
